# A Database of Snow on Sea Ice in the Central Arctic Collected during the MOSAiC expedition

**DOI:** 10.1038/s41597-023-02273-1

**Published:** 2023-06-22

**Authors:** Amy R. Macfarlane, Martin Schneebeli, Ruzica Dadic, Aikaterini Tavri, Antonia Immerz, Chris Polashenski, Daniela Krampe, David Clemens-Sewall, David N. Wagner, Donald K. Perovich, Hannula Henna-Reetta, Ian Raphael, Ilkka Matero, Julia Regnery, Madison M. Smith, Marcel Nicolaus, Matthias Jaggi, Marc Oggier, Melinda A. Webster, Michael Lehning, Nikolai Kolabutin, Polona Itkin, Reza Naderpour, Roberta Pirazzini, Stefan Hämmerle, Stefanie Arndt, Steven Fons

**Affiliations:** 1 WSL Institute for Snow and Avalanche Research SLF, Davos Dorf, Switzerland; 2 Victoria University of Wellington, Wellington, New Zealand; 3 Department of Geography, University of Victoria, Victoria, BC Canada; 4 Alfred-Wegener-Institut Helmholtz-Zenütrum fr Polar und Meeresforschung, Bremerhaven, Germany; 5 Thayer School of Engineering at Dartmouth College, Hanover, New Hampshire USA; 6 CRYOS, School of Architecture, Civil and Environmental Engineering, EPFL, Lausanne, Switzerland; 7 Finnish Meteorological Institute, Helsinki, Finland; 8 Svalbard Integrated Arctic Earth Observing System, P.O. Box 156, 9171 Longyearbyen, Norway; 9 Woods Hole Oceanographic Institution, Woods Hole, Massachusetts USA; 10 International Arctic Research Center, University of Alaska Fairbanks, Fairbanks, USA; 11 Arctic and Antarctic Research Institute, AARI, Saint-Petersburg, Russia; 12 UiT The Arctic University of Norway, Tromsø, Norway; 13 SCANCO medical AG, Wangen-Brüttisellen, Switzerland; 14 Cryospheric Sciences Lab, NASA Goddard Space Flight Center, Greenbelt, Maryland USA; 15 Earth System Science Interdisciplinary Center, University of Maryland, College Park, Maryland USA

**Keywords:** Cryospheric science, Techniques and instrumentation

## Abstract

Snow plays an essential role in the Arctic as the interface between the sea ice and the atmosphere. Optical properties, thermal conductivity and mass distribution are critical to understanding the complex Arctic sea ice system’s energy balance and mass distribution. By conducting measurements from October 2019 to September 2020 on the Multidisciplinary drifting Observatory for the Study of Arctic Climate (MOSAiC) expedition, we have produced a dataset capturing the year-long evolution of the physical properties of the snow and surface scattering layer, a highly porous surface layer on Arctic sea ice that evolves due to preferential melt at the ice grain boundaries. The dataset includes measurements of snow during MOSAiC. Measurements included profiles of depth, density, temperature, snow water equivalent, penetration resistance, stable water isotope, salinity and microcomputer tomography samples. Most snowpit sites were visited and measured weekly to capture the temporal evolution of the physical properties of snow. The compiled dataset includes 576 snowpits and describes snow conditions during the MOSAiC expedition.

## Background & Summary

Snow cover modulates the thermal and optical properties of the sea ice surface and the energy fluxes between the ocean and the atmosphere, directly impacting the amount of ice growth in the winter and ice melt in the summer^1–10^. Despite its importance, measurements of the physical properties of snow on sea ice throughout the annual cycle are limited to just a few expeditions (e.g. SHEBA^11^, N-ICE^12^, TARA^13^, Russian drifting stations^14^) and the Canadian Arctic (See Table 1* in^15^). Due to the rapid changes in the Arctic, data in this region quickly becomes outdated. As a result, this Multidisciplinary drifting Observatory for the Study of Arctic Climate (MOSAiC) dataset has increased value due to its recent collection (compared to SHEBA), and as a result, is likely more representative of the new Arctic stricken by climate change. The previous lack of up-to-date regional data causes biases in model representations of sea ice variables^16^ and significant uncertainty in how sea ice influences the global energy budget. The IPCC 2019 Special Report on the Ocean and Cryosphere in a Changing Climate^17^ identifies snow on sea ice as one of the “key knowledge gaps and uncertainties“ limiting predictive climate models. In addition to its major implications on the physical properties of sea ice in winter, the snowmelt in summer acts as a freshwater source affecting melt ponds and upper ocean stratification and determines light and nutrient availability for polar marine ecosystems^18^. As the snow melts, bare ice is exposed. Preferential melting of grain boundaries in columnar ice produces the surface scattering layer (SSL): a granular, snow-like material that behaves similarly to meteoric snow in certain respects^19^. Understanding the physical properties of the SSL is key to understanding sea ice albedo and surface ablation.

This dataset documents the stratigraphy and microstructure of the snow cover and, in the absence of snow, the microstructure of the SSL and ice surface throughout the MOSAiC expedition^20^. This dataset and data paper detail all measurements categorised as “snowpit events“ during MOSAiC. Each snowpit “event“ corresponds to one visit to a snowpit location and has an assigned unique device operation ID. The dataset documents the temporal and spatial evolution of the physical properties of the snow/ice surface layer. Expected applications of these data include snow-focused and interdisciplinary research areas, such as (1) thermal conductivity of snow on sea ice and thermal transfer across the ocean-ice-atmosphere system; (2) surface energy budget and radiative transfer through the snow and ice column into the upper ocean; (3) satellite retrievals of snow and ice thickness; (4) the freshwater budget. We used 16 different instruments to characterise the physical properties of snow and the SSL during MOSAiC.

## Methods

**Fig. 1:**
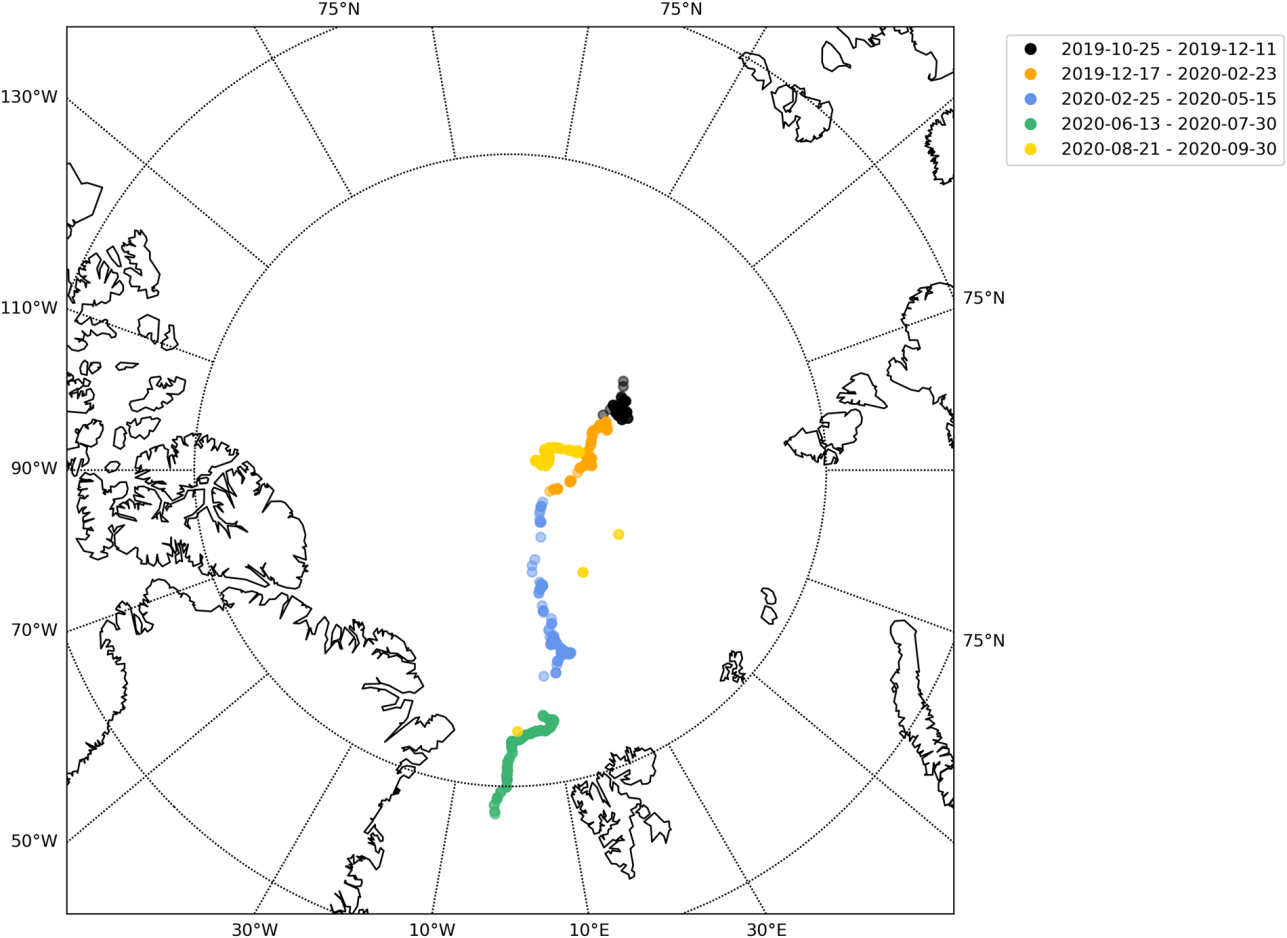
Snowpit locations of each unique device operation ID. A map showing the latitude and longitude of each snowpit visit from 2019-10-25 to 2020-09-30. Each device operation ID is indicated by one mark on the figure and the colours represent the time period for each device operation ID beginning with PS122/1, PS122/2, PS122/3, PS122/4 and PS122/5 respectively. Refer to the usage notes to relate the device operation ID to the dates of interest and the contact person. The marks have transparency so the darker marks represent multiple measurements in one coordinate region.

**Fig. 2:**
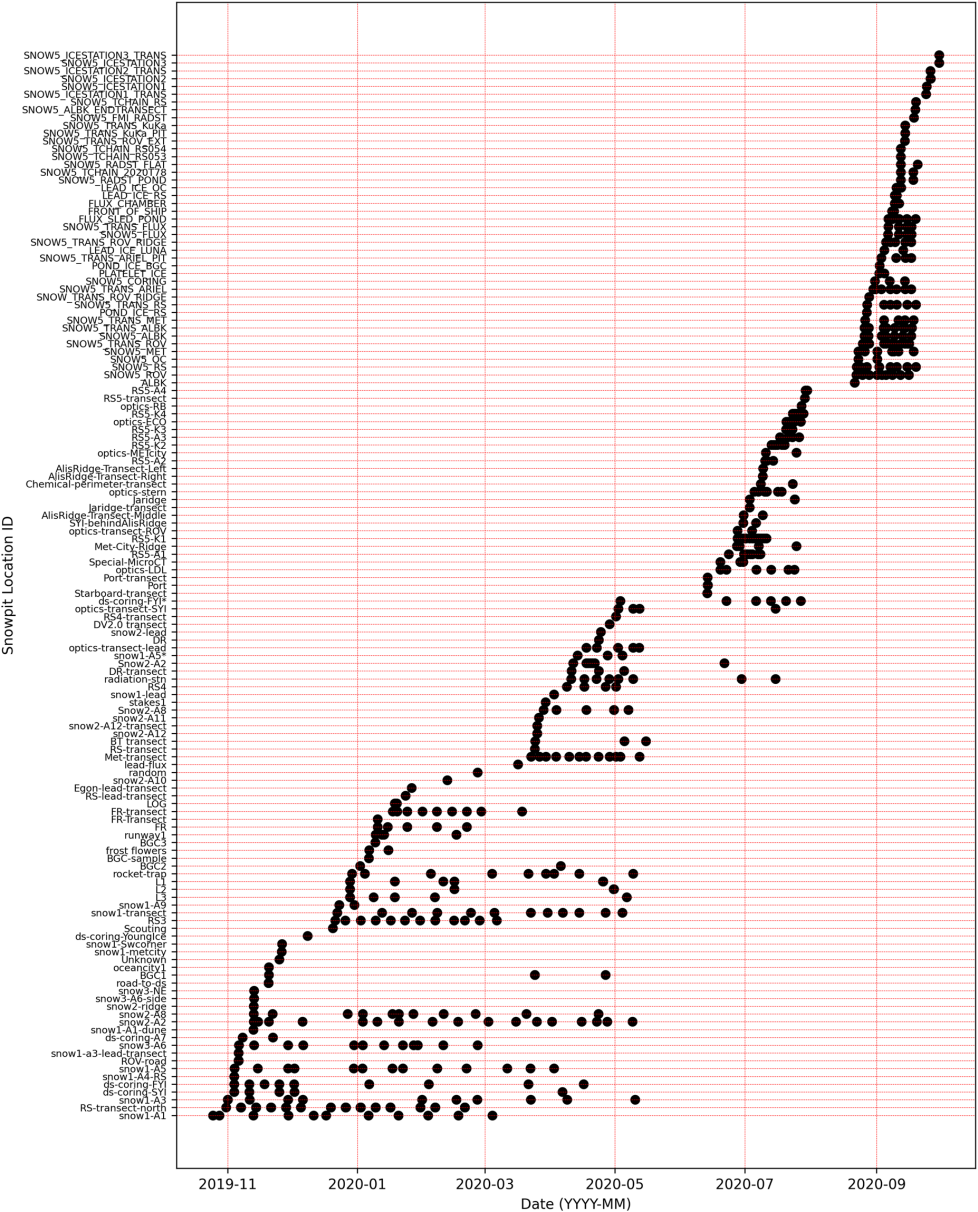
Time series of snowpit measurements at each snowpit site. A black mark indicates one visit to the snowpit site. The name of the snowpit site is indicated on the y-axis. The relocation of the central observatory and ice dynamics can be seen through the discontinuation of certain time series. This figure is to visualise the overall snowpit time series. However, due to the limited font size, please refer to the metadata publication^38^ for detailed information on specific dates of the snowpit site visits.

The study area was on drifting sea ice, originally located 85.44 degrees North. The locations of the snowpit sites are shown in Fig. 1. This dataset’s difficult-to-access latitudinal range, unprecedented detail and wide range of parameters measured make it a unique dataset for studying the role of snow in the Arctic sea ice system. Observations were conducted in two primary modes to account for temporal and spatial heterogeneity. The first mode aimed to collect a time series of measurements at points of interest by setting up designated, undisturbed areas in the central observatories (CO), approximately 0.01–0.05 km^2^, as “clean” snow areas, where we measured adjacent snowpits at least weekly at snow pit sites to create a time series of the metamorphosing snowpack. The snowpit time series can be seen in Fig. 2. The second mode consisted of linear transects on multiple ice types and topographies for sampling snow heterogeneity. More information can be found in section 3.4. Occasionally we conducted one-time measurements at sites of specific interest, such as newly-formed leads, refrozen ponds, and with remote sensing or albedo transects.

**Fig. 3:**
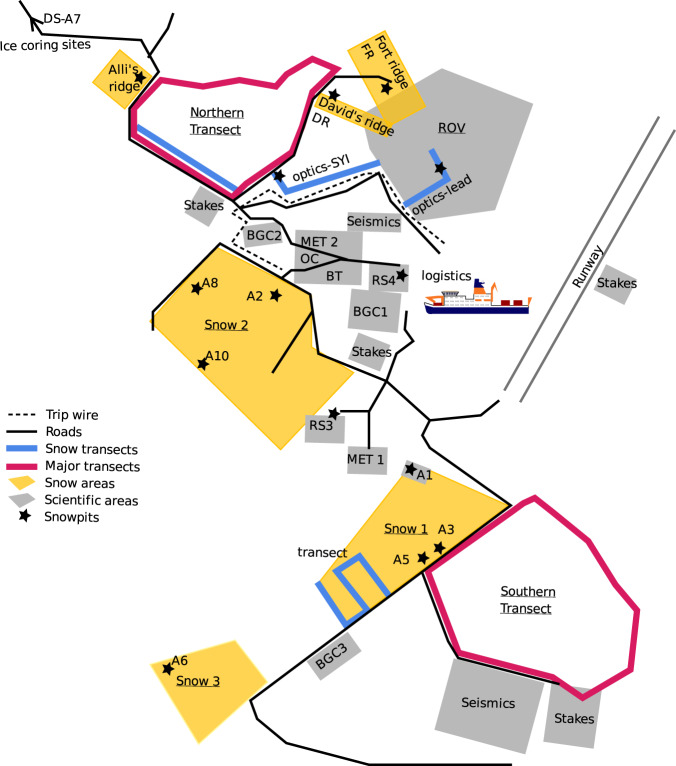
Schematic diagram of the snowpit locations across central observatory 1. Schematic diagram of central observatory 1 (CO1) adapted from the maps used during the expedition from 2019-10-25 - 2020-05-15. For detailed information on location acronyms, please refer to the snow and ice overview manuscript^20^.

**Fig. 4:**
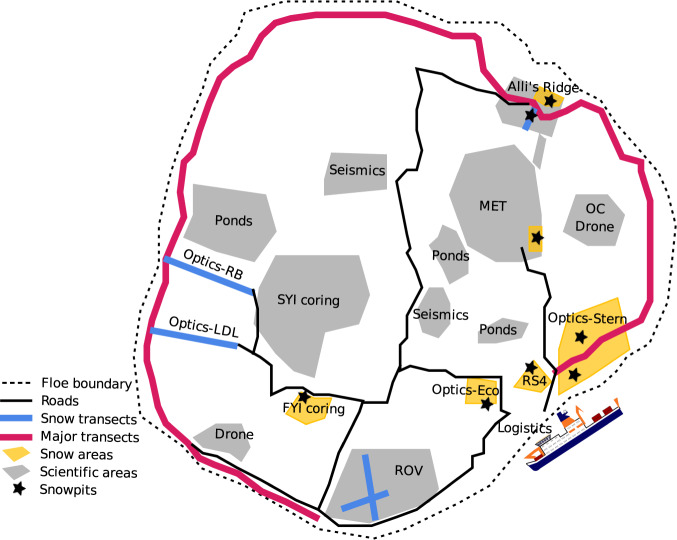
Schematic diagram of the snowpit locations across central observatory 2. Schematic diagram of central observatory 2 (CO2) adapted from the maps used during the expedition from 2020-06-13 - 2020-07-30. For detailed information on location acronyms, please refer to the snow and ice overview manuscript^20^.

**Fig. 5:**
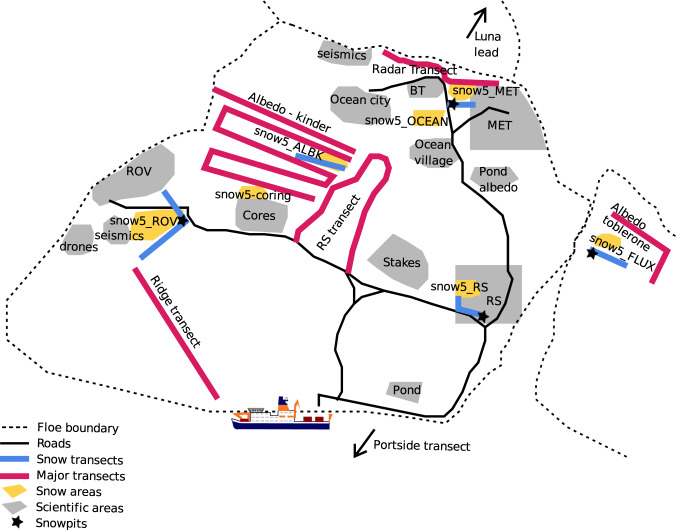
Schematic diagram of the snowpit locations across central observatory 3. Schematic diagram of central observatory 3 (CO3) adapted from the maps used during the expedition from 2020-08-21 - 2020-09-30 after the relocation of Polarstern. For detailed information on location acronyms, please refer to the snow and ice overview manuscript^20^.

The MOSAiC expedition transitioned between three ice floes^20^ (CO1, CO2 and CO3). Figures 3–5 show locations of snowpit sites on these ice floes during the MOSAiC expedition. Having three separate floes produced a discontinuity in the snowpit time series; more details can be found in section 3.4. Further discontinuity in time series was due to the highly dynamic nature of sea ice, and we often had to relocate some snowpit sites. Despite this, we could obtain measurements of the ice surface and snow stratigraphy throughout the year. The time series of visits to each snowpit site can be seen in Fig. 2.

**Fig. 6:**
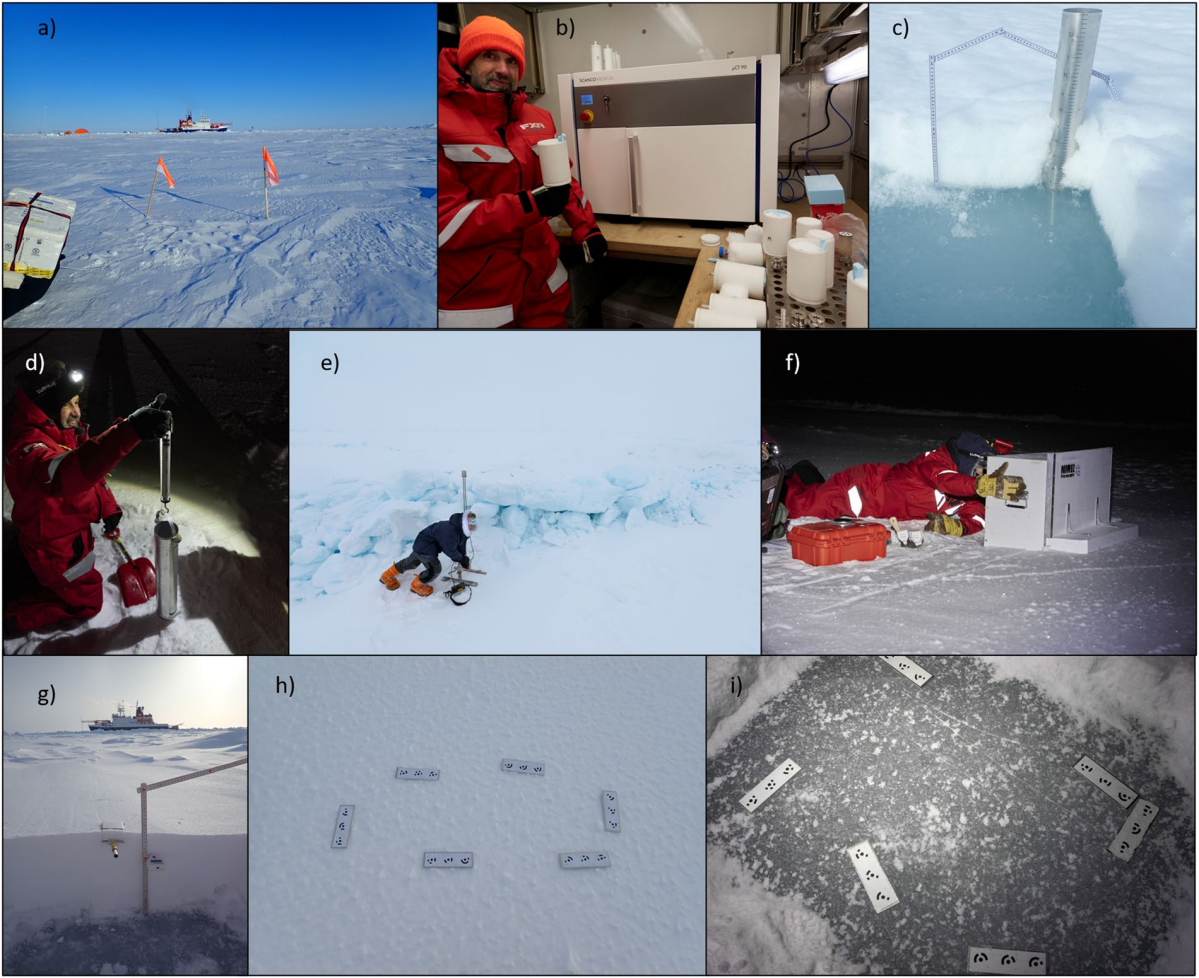
A combination of the different instruments taken to the snowpit site. (**a**) An example overview picture^45^, photo credits with publishing permission: A. Macfarlane. (**b**) The micro-CT^53^ mounted in the cold laboratory on Polarstern. A snow sample is being held in the white sample holder of 88 mm diameter; other sizes of samples can be seen in the table on the right side of the image, photo credits with publishing permission M. Jaggi. (**c**) The SWE tube^43^ and the ruler^49^ in the snowpit in the spring season, photo credits with publishing permission: A. Macfarlane. (**d**) The SWE tube^43^ in action in the field, photo credits with publishing permission: M. Jaggi. (**e**) The SMP^40^ measuring penetration resistance in front of an ice ridge. photo credits with publishing permission: D. Ruché. (**f**) The NIRbox^41^ taking an image of the snowpit wall, photo credits with publishing permission: M. Jaggi. (**g**) A density cutter^48^ (left of the ruler) and thermometer^44^ (right of the ruler) inserted in the snowpit wall, photo credits with publishing permission: A. Macfarlane. (**h**) An SfM example image^46^ showing the SfM targets placed on the naturally illuminated snow surface, photo credits with publishing permission: A. Macfarlane. (**i**) An SfM example image^46^ showing the SfM targets placed on the snow-ice interface; this image is illuminated using a head torch in the field, photo credits with publishing permission: M. Schneebeli.

**Fig. 7:**
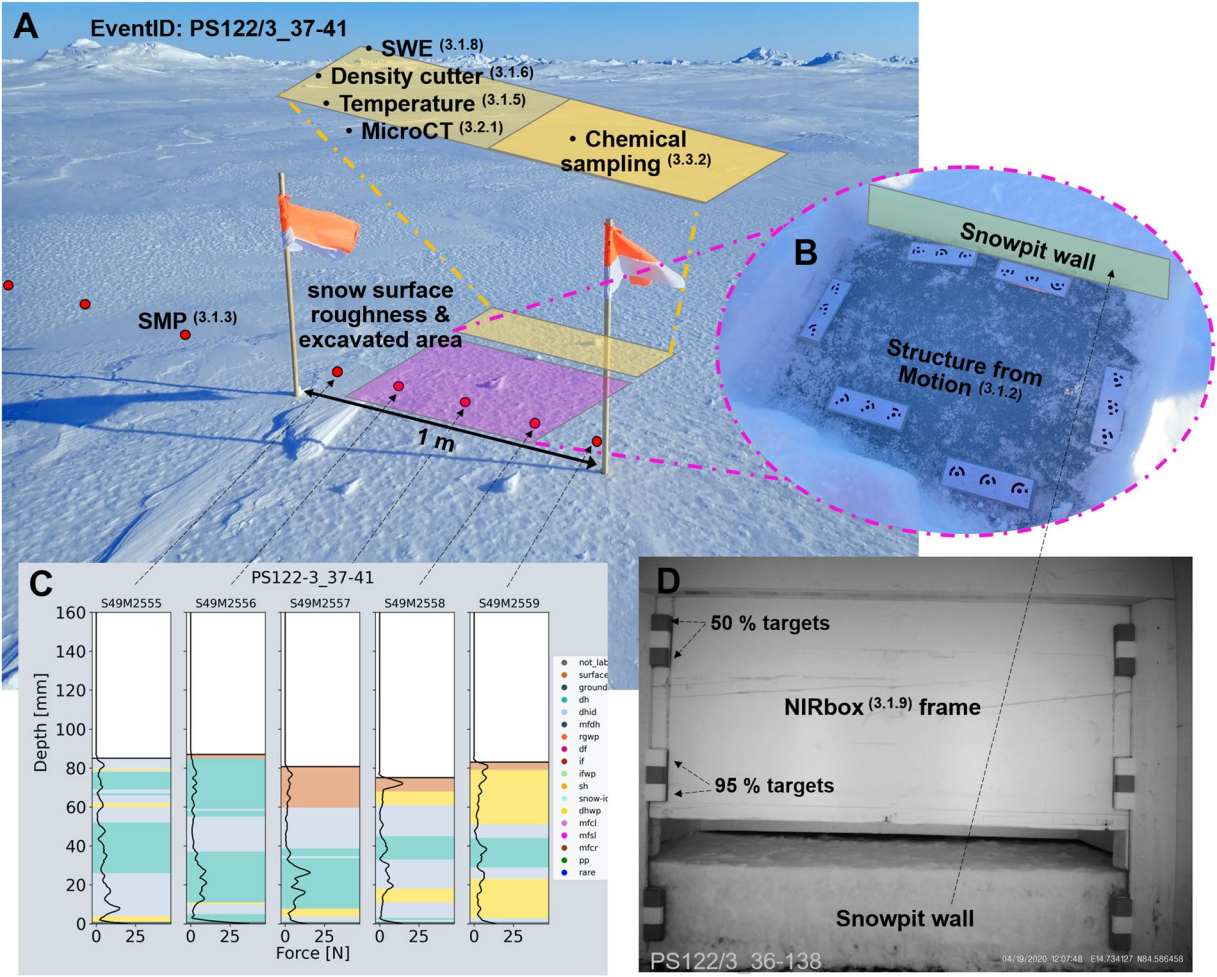
A case study of the measurements taken during event ID PS122/3_37-41. The overview image in the background of (**A**) gives an example of the conditions upon arrival at the snowpit. Annotations to this image show the different measurement locations and their relation to each other. The pink highlighted box shows the surface roughness measurement (SfM) location and the snowpit excavation area to allow access to the snowpit wall. Once excavated, the yellow box shows where the core measurements are taken, listed as bullet points. (**B**) shows the excavated pit revealing the underlying sea ice surface, also measured for roughness using SfM. The red points indicate the SMP measurements. The five central SMP measurements are located in the snowpit, and to capture spatial heterogeneity, sometimes additional measurements were conducted to the left and right of the snowpit. (**C**) shows the SMP force signals over depth with the categorised grain types^26^. This gives an indication of the spatial heterogeneity within the snowpit. The image in (**D**) is from the NIRbox during device operation ID PS122/3_36-138. The annotations of this figure show the reference targets (95 and 50%) and the NIRbox frame above the excavated snowpit wall.

A total of 16 different instruments were used at the snowpit sites during MOSAiC (details of these instruments can be found in Table 1 and images of each instrument can be seen in Fig. 6). A standard operating procedure manual (SOP) for each instrument was written to increase continuity between operators (see supplementary material). We created standardised field protocols for three types of snowpits (from protocol A, including all possible observations, to protocol C, which should be followed when there were time (or other) limitations). The protocols indicated the order of measurements at each snowpit. Table 2 provides details of each device used for protocols A, B and C. However, it is important to note that the A, B, and C protocols were not strictly followed throughout the expedition. The final set of instruments depended on the time and ice conditions on the given day. Figure 7A shows an example of the snowpit layout, and details of each measurement are given in section 3.1. Orientation of the snowpit was wind dependent in the winter when there was high wind/induced mixing of the snow cover. The snowpit was dug with the operator facing the wind to reduce snow accumulation in the pit and reduce contamination of the chemical samples. When the sun appeared over the horizon, the snowpit was dug towards the sun to keep the snowpit wall in the shade.

**Table 1:** Device details.

Instruments	Device Type	Number of devices	Measurement location	Measurement Parameters Raw	Extracted Parameters after processing	Ref
General observations	Protocol sheets	1	*In-situ*	Snow height, weather conditions, surface type	snow height (cm), weather conditions, surface type	^38,39,42^
Micro-CT	X-ray computer tomograph	1	Cold lab on Polarstern	3-D geometry	Stratigraphy, Microstructure: Porosity, Density (kg m^−3^), Connectivity, SSA (m^2^ kg^−1^), Anisotropy, Thermal conductivity (W m^−1^ K^−1^)	^53^
Micro-CT	DEP Snow Casting Sampler	1	*In-situ* for later processing	sample extraction for 3-D geometry	Stratigraphy, Microstructure: Porosity, Density (kg m^−3^, Connectivity, SSA (m^2^ kg^−1^), Anisotropy, Thermal conductivity (W m^−1^ K^−1^)	^53^
snow micro penetrometer (SMP)	snow micro penetrometer 49, 43, 31	3	*In-situ*	Hardness	Density (kg m^−3^), SSA (m^2^ kg^−1^), grain type^26^, stratigraphy	^40^
NIR camera	Box with Near-Infrared camera at 850 + 950 nm	1	*In-situ*	Images of NIR reflectance at 850 + 940 nm	SSA (m^2^ kg^−1^), stratigraphy	^41^
ETH-SWE	ETH SWE tube	1	*In-situ*	SWE	SWE (mm)	^43^
Temperature	Snow Temperature 1, 2, 3, 4, 6	5	*In-situ*	Temperature	Temperature (°C)	^44^
Density cutter	Emerald500 electronic scales 1–4 and 100 cm^3^ density cutter	4	*In-situ*	Weight of density cutter + snow	Density (kg m^−3^)	^48^
GPS	Garmin GPS	1	*In-situ*	GPS coordinates	GPS coordinates (lat/lon)	^50^
Stable water isotopes	Stable water isotopes, vials	NaN	*In-situ* sampling, WSL laboratory processing	sample extraction (melted)	δ^18^O, Deuterium (ratio)	^52^
Overview pictures	Overview pictures, Olympus GP-5	1	*In-situ*	Images	Overview Images	^45^
SfM pictures	SfM picture sets, Olympus GP-5+ targets	1	*In-situ*	Image sets	Small scale DEMs, surface roughness	^46^
Ruler	Ruler	1	*In-situ*	Snow height + position of other measurements	Snow height + position of other measurements (cm)	^49^
Salinity	YSI 30 Salinity, Conductivity, Temperature	1	Polarstern dry laboratory	Salinity, Temperature	Salinity (ppt)	^51^
Permittivity	POGO portable soil sensor, Permittivity	1	*In-situ*	Dielectric permittivity (real + imaginary)	Liquid water content (g m^−^^3^)	^47^

Information about each device taken into the field can be found in this table. It gives details on the device type, the published dataset related to this instrument (Ref), the number of devices used in the field, where the measurement took place, the parameters obtained directly from the raw dataset and the possible parameters that can be extracted from this dataset.

**Table 2:** Protocol Steps.

Protocol A (Complete sampling of physical and chemical properties)	Protocol B (Typically only the physical measurements taken)	Protocol C (Quick measurements)
*All protocol B and C measurements	*All protocol C measurements	Metadata collection
Chemical Sampling	Overview pictures of the site	Snow Micro Penetrometer profiles (SMP)
	Pictures for airborne structure from motion (SfM)	Near infrared pictures (850 nm and 940 nm)
	GPS waypoint	
	Snow height	
	SWE (ETH tube)	
	Density (volumetric)	
	Snow temperature	
	micro-CT samples/ casting	
	Salinity and stable water isotope samples	
	Permittivity	

This table lists the instruments taken to each of the snowpit types; “A“, “B“ and “C“. This was not always followed directly due to time, conditions and other limitations.

Each snowpit “event” corresponds to one site visit and has an assigned unique device operation ID. The equipment needed for the snowpit measurements was taken in a sledge to the snowpit site marked with flags. The flags allowed us to return to the exact location of the previous measurement. Most measurements were conducted *in situ* (see Table 1). Other measurements were conducted in laboratories onboard Polarstern or shipped to laboratories after the expedition ended. For *in-situ* measurements, the first step when arriving at the snowpit site was to take an overview image, as seen in Figs. 6a, 7a, which provides information on the general conditions. Annotations on Fig. 7A show the different measurement locations and their relation to each other. The second step was to conduct the nondestructive measurements that require an undisturbed state of the snow at the snowpit site, including the snow surface structure from motion images (SfM, see Fig. 6h) and the snow micro penetrometer (SMP) measurements (Fig. 6e). The five central SMP measurements are located in the snowpit (the red-filled circles, Fig. 7A) and additional SMP measurements were conducted on both sides of the snowpit to capture spatial heterogeneity. After the SMP measurements, the pit was then excavated (area highlighted in pink in Fig. 7A), allowing access to the snowpit wall and the sea ice surface. Once the snowpit wall was exposed, a near-infrared (NIR) camera mounted in a light-proof box (NIRbox) was used to image the flat snowpit wall (the NIRbox is seen in Fig. 6f and an example of a NIRbox image is seen in Fig. 7B), and the height of the snowpit was recorded with the ruler (Fig. 6c). The relief of the sea ice surface was also measured using SfM (Fig. 6i). The measurements which were “destructive” could then commence. The yellow highlighted box in Fig. 7A shows where the core destructive measurements were taken, listed as bullet points in Fig. 7A and details can be found in section 3.1. Images of these measurements can be seen in Fig. 6b–d,g.

Throughout this data paper, a depth of 0 mm represents the snow-ice interface. When measuring the SSL in the melt season, a depth of 0 mm represents the ice surface.

### *In situ* measurements

#### Overview pictures

We used a standard digital camera (Olympus tough TG-5) to document the surface conditions and larger area for each snowpit site visit on arrival. Figure 6a shows an example image.

#### Structure from motion images (SfM)

The millimetre-scale surface roughness is important for the scattering of shortwave visible^21^, near-infrared and higher-frequency microwave radiation. We used a standard digital camera (Olympus Tough TG-5) and a set of unique reference targets to take sets of images. These images are processed using the structure-from-motion method (SfM)^22^ into high-resolution, small-scale (approximately 0.5 m × 0.7 m) digital elevation models (DEMs) to estimate the roughness of the snow surface and the snow-ice interface. The unique reference targets were printed on never tear paper and glued onto metal plates. We distributed the reference targets around a small (Approximately 0.6 m × 0.6 m) area and took pictures from different angles. We included all targets in each image, ideally overlapping by at least 80%. We took pictures with the maximum wide angle of the camera. We used a headlamp to illuminate the scene during the polar night. The illumination was kept constant during the measurement. We took two sets of images; the first of the surface before excavating the snowpit (Fig. 6h) and the second of the snow-ice interface after the snowpit excavation and using a fine-haired brush, to remove all the remaining snow off the snow-ice interface. (Fig. 6i).

#### Snow micro penetrometer

The snow micro penetrometer (SMP) is a portable device for measuring high-resolution vertical profiles of snow penetration resistance in the field. The penetration resistance can be correlated to snow microstructure^23,24^. The penetration force is measured using a piezo-electric sensor and digitally recorded every 4 micrometres. The SMP signal can be analysed to infer stratigraphy, snow type, and snow microstructure at 1–4 millimetres vertical resolution. Snow density^25^ and specific surface area (SSA)^23^ can be estimated from the force signal. Through repeated measurements along transects, the SMP can help relate detailed point-scale snow profiles to a more extensive sampling area and provide information about the spatial heterogeneity of snow stratigraphy and physical snow properties. We used the SMP in snowpits and along the transects before excavating the profile wall. The SMP was taken to 389 snowpits and transects. This dataset consists of 6837 penetration resistance profiles. Figure 6e shows the SMP measuring a ridge, and the inset in the bottom left of Fig. 7 shows the SMP force signals with the categorised snow grain types^26^. This dataset should be used with caution in the summer season when the snow is melting as the sensor is not adapted to measuring in these conditions.

#### Snowpit height

This parameter is measured in centimetres. We used a foldable wooden ruler to measure the height of the snow and the depth of the SSL. We used the ruler to reference the height of temperature measurements, density measurements, salinity profiles and chemical sampling. The set-up of the ruler can be seen in Fig. 6c,g.

#### Temperature

The snow temperature was measured using a waterproof thermometer with a needle probe using a ruler to determine the measurement height. Every snowpit included a surface and snow-interface temperature measurement. More measurements are taken at 5-cm intervals, starting at the bottom. Multiple thermometers were used throughout the expedition and calibrated before departure to ensure they were measuring accurately. The thermometer can be seen in Fig. 6g after inserting it into the snowpit wall.

#### Density cutter

We used a density cutter of fixed volume (100 cm^3^) and known weight to measure the density of snow/SSL in 3 cm intervals^27^. We recorded the height of the sampled volume with a ruler and the combined weight of the density cutter and the snow inside with digital scales. The resulting density is the weight of snow/volume. The snow from the density cutter was collected in plastic containers and used for subsequent salinity and stable water isotope measurements.

#### Complex dielectric permittivity *ε* (real and imaginary components)

Dielectric permittivity (***É***) and dielectric loss (***Ë***) measurements were made of discrete snow layers and at fixed vertical intervals at the remote sensing site during the summer months (2020-06 - 2020-09). We measured variables using the Stevens Water Monitoring Systems Hydra Probe (a.k.a. hydraprobe)^28^. The hydraprobe consists of a central waveguide and three outer rods, each 4.5 cm in length and 3 mm wide, to measure the sample’s impedance at 50 MHz over a cylindrical area of 5.7 cm in length by 3 cm in diameter^28^. The sensor was calibrated using isopropyl alcohol for ***É***(±0.6%) and a saline solution of known conductivity for *Ë*(±0.7%)^29^. Other examples of the use of this sensor in snow and sea ice studies include Backstrom and Eicken (2006)^30^ and Scharien *et al*.^31^. Measurements were obtained by horizontally inserting the probe into the snow, at a given layer/interval (every 3 cm), to their maximum depth.

#### Snow water equivalent

We measured the weight and volume of a snow sample using an aluminium SWE ETH-tube to calculate the snow water equivalent (SWE). SWE is the amount of water contained within the snowpack and hence the water depth that would theoretically result if the entire snowpack melted instantaneously. Snow height (HS) is related to SWE by the local bulk density of snow (*ρ*_*b*_) using the equation SWE = HS *ρ*_*b*_/*ρ*_*w*_, where HS is in millimetres, (*ρ*_*b*_ is in kg m^−3^, *ρ*_*w*_ is the density of water (1000 kg m^−3^), and SWE is in millimetres of water. At each snowpit site, the ETH-SWE tube of length 55 cm and inside surface area of 70 cm^2^ was inserted into the snow vertically and then closed off at the bottom. The weight of the cylindrical tube was then measured with a spring scale that was calibrated specifically for the cross-section and weight of this tube. The spring in the spring scale was not affected by cold temperatures. If the snow cover was deeper than 0.45 m (height of the tube), then the SWE was measured in several steps, using a ruler to note the snow height range from which the sample was taken. This was often the case for snowpits in ridged areas. The total water equivalent of the snow cover was then calculated as the sum of the water equivalent (WE) of the vertically aligned samples. We also measured the height of snow in each measurement, so we could back-calculate the snow density using an independent method.

#### Near infrared reflectance images

This instrument measures the snow/SSL surface and snow profile wall’s near-infrared (NIR) reflectivity. A NIR MAPIR camera (Survey3N Camera)^32^ was built into a lightproof box and triggered by an external button. We named this instrument the NIRbox, and it can be used horizontally looking at the snowpit wall or vertically looking down at the snow/ice surface. The processing of the NIRbox images must consider the sensitivity of the different colour pixels. The setup of the reference targets, the flat field and the diffuse illumination are crucial to getting high-quality images. A geometrically corrected NIR photo objectively measures the snow stratigraphy and is observer-independent. This efficient measurement has been adapted for polar night and day by blocking out external sunlight and packaging the camera and the illuminating infrared lights into a wooden box. The length and height of the inside of the box are 500 mm × 675 mm. We used a blanket during polar summer to prevent light entering the box. As an extra precaution, a dark image (without any lamps) was taken for each image set, followed by images with each of the two lamps (using external light switches) to account for potential light leaks. Lambertian reflectance targets of 95% and 50% were mounted inside the box to account for irregular light conditions. Lamps with two different wavelengths, 850 nm and 940 nm, were mounted inside the box. We can use the images obtained horizontally by the MAPIR camera in an excavated snowpit to identify layers of snow grains with different SSA with a spatial resolution of about 1 mm^33^. This approach can highlight the snow stratigraphy’s vertical and horizontal spatial heterogeneity. If the snowpit depth exceeded the NIRbox height, measurements were repeated for different heights, using an object or feature as a reference. NIR was also used vertically facing down to take images of the surface to account for the small-scale spatial heterogeneity of the SSA of the snow or SSL surface.

### Ship-based measurements

#### Micro computer tomography

Cylindrical snow and ice samples of radii 44 mm, 68 mm, 88 mm and a maximum height of 110 mm were taken from the field using a cylindrical drill and transported to the ship in an insulated container. We scanned the samples within 24 hours using a desktop cone-beam micro-CT90 (Micro-CT)^34^ set-up in a −15 °C cold laboratory.

#### Salinity

The salinity of melted snow samples was measured using the YSI 30 Salinity, Conductivity and Temperature sensor^35^. The snow was collected using the density cutter in the field and then melted and measured in the laboratory on Polarstern. The transport containers, as well as the YSI tip, were cleaned using milli-Q water. Salinity was measured at the same vertical intervals as density.

### Shore-based lab measurements

#### Oxygen and hydrogen isotopes

After the salinity measurement, a small glass vial was filled with the melted snow water and transported to the Swiss Federal Institute for Forest, Snow and Landscape Research, WSL, to analyse the stable water isotope ratios.

### Sampling strategy

The winter snow accumulation on sea ice is highly heterogeneous due to (1) wind-driven snow redistribution, (2) dynamic local topography and (3) differences in underlying ice types and thicknesses. Changes in local topography are mainly caused by dynamic processes like ridge and lead formation and existing refrozen ponds. Topography influences snow accumulation by modifying local wind fields, which can affect snow erosion and deposition. Differences in underlying ice type/thickness are caused by sea ice history (e.g. seasonal ice vs multiyear ice or melt pond history), dynamic processes of ridge formation, and the refreezing of leads. The physical properties and thickness of the underlying ice, in addition to atmospheric conditions, alter the temperature gradients across the snowpack, which impact the evolution of snow microstructure and snow physical properties in return. The combination of spatially variable snow deposition and erosion with spatially variable metamorphism led to high spatial heterogeneity over small horizontal scales. Two snowpit sites just decimeters apart often show significant differences in stratigraphic sequence and microstructure. Therefore, a single vertical profile is not necessarily representative of the snowpack, and the time series of snowpit observations taken adjacent to one another conflate spatial heterogeneity and temporal variability, making it challenging to assess the evolution of the snowpack. We chose a range of sites for snow measurements to understand the spatial heterogeneity of snow and the SSL. The locations of the snowpit sites included level seasonal ice, level multiyear ice, ridged areas, and refrozen leads. To account for spatial heterogeneity in the winter season, we collected high-resolution vertical profiles of penetration resistance along short transects (1.5–5 m) on either side of the snowpit site (see the coloured red circles in Fig. 7), to upscale the detailed snow profile measurements from within the snowpit itself. These measurements were part of snowpit protocols A, B and C. See Table 2 for more details. In certain instances, we also collected additional extended transects to account for larger-scale spatial heterogeneity on multiple ice types and topographies. These larger transects often consisted of snow micro penetrometer (SMP) measurements but were also co-located with magnaprobe transects. Magnaprobe transects are excluded from this data paper, but further details can be found at Itkin (2020)^36^, alongside the table where individual snow pit events can be related to the individual Magnaprobe transect events named “mosaic-transect-actionlog-updated.xlsx”^36^. Snow and magnaprobe transects covered a larger area than the snowpit sites, sampling at predefined intervals depending on the area of interest and the study. In summer, the spatial heterogeneity of the surface layer was caused by different ice types and topographic features and winter wind-driven snow redistribution to the ridges persisting into the summer season. Sampling strategies for summer and winter were similar.

Ice deformation during the expedition cut off access to some snowpit sites and disrupted the snowpit time series. We accounted for these time-series disruptions by sampling more snowpit sites at the start of the expedition in case some of them became inaccessible. It is also important to mention that documenting the snow distribution around these dynamic events is valuable information, so these topographic features were also a focus when choosing the sampling sites. For example;

- The occurrence of leads in the field site can obstruct an existing site and prevent further measurements. However, once they refreeze, such leads allow the investigation of the snowpack over a newly formed lead, where snow metamorphism occurs more rapidly due to the relatively higher heat flux through thinner ice. By studying leads, we can follow the accumulation process from the start of sea ice formation.
- Ridging of the ice in the vicinity of a snowpit site often caused the site to become inaccessible and drastically changed the snowpack in the surrounding area. The snowpack near ridges was deeper due to wind-driven snow redistribution into drifts around ridges. Ridges add roughness to the topography, decreasing local wind speed and increasing local snow accumulation. Unless the ridge obstructed the snowpit site, measurements continued and showed how ridges affect the evolution of snowpack.

More snow-relevant events which are captured in this dataset include:

- Snow redistribution by wind
- Rain on snow
- Warm air intrusions
- Snowfall
- Melt-freeze cycles
- Surface hoar
- Melt ponds

Detailed information on the drift tracks^37^ and the different COs can be found in the ICE overview publication^20^. Information on the time series continuity between CO1, CO2 and CO3 is as follows (each period corresponds to a different group of scientists on board):

- **2019-10-25 - 2019-12-11**- The setup of the CO1 and choosing snowpit sites for the start of many snowpit time series.
- **2019-12-17 - 2020-02-23**- The start of many time series and continuation of time series on CO1.
- **2020-02-25 - 2020-05-15**- Large ice dynamics resulted in the discontinuity of some time series.
- **2020-06-13 - 2020-07-30**- Re-location of the research vessel to CO2. On the way to CO2, two measurements were conducted en route. Start of many time series and the continuation of one time series at first-year ice (FYI) coring site until the end of CO2’s life cycle.
- **2020-08-21 - 2020-09-30**- Re-location north to CO3. This is the start of many time series with no continuation of previous time series. Measurements in 3 locations were conducted on the way back to Bremerhaven.

## Data Records

### Overview of datasets

The corresponding data to this publication can be found in the snowpit raw dataset bundle^38^. The bundle includes all data collected from instruments taken to the snowpit sites and all metadata linked to the device operation ID. Within the snowpit dataset bundle are the following datasets:

- Snowpit metadata TXT files^39^. Each event contains a text file in the metadata dataset, which explains the event, attendees, weather conditions, instruments used and samples taken. The metadata file details what is not easily visible in the data. It gives an overview of conditions at the snowpit site, who worked on it, features of the surrounding landscape, and conducted measurements and samples. It makes it much easier to reconstruct the circumstances during the measurements.
- Snowpit SMP force profiles^40^. The measurements in this data publication are grouped by event. One event corresponds to one trip to the ice and often includes multiple SMP measurements; see Fig. 7. The Location column gives information about where the trip took place. See the maps in Figs. 3–5 for details. The ID column gives an internal location of each measurement with respect to the snowpit. See “Further details: SnowMicroPen raw data: details and explanations of acronyms“ in the Pangaea publication^40^. This can also be accessed using the link: https://download.pangaea.de/reference/109819/attachments/details.pdf.
- Snowpit near-infrared (NIR) images^41^. Uploaded photos from the MAPIR NIR camera in both jpeg and raw format. The MAPIR software can be used to create TIFF files for further analysis. Details of the wavelength and location of each NIR image can be found in the published table alongside the dataset.
- Snowpit surface type^42^. This table provides information about the snow surface on arrival at the snowpit site, it contains several possible snow surface types. It is important to note that the different observers throughout the expedition completed this table subjectively. This table should not be used for detailed analysis, only to obtain an idea of the conditions at the time of the event.
- Snowpit snow water equivalent^43^. The SWE and snow height are recorded in the metadata spreadsheet file for SWE measurements. The column MeanRho is calculated automatically. If the snowpit was variable or there was a lot of snow, there would be several SWE measurements for one snowpit visit. These measurements are listed in the table with the same device operation ID. Please see the comments to see if the whole snow profile or only a specific layer, like new snow, was measured.
- Snowpit temperature profiles^44^. The temperature device operation ID can be found in the first column alongside the corresponding snow height at which the temperature was measured. Temperature is recorded in Celsius in the column “Temperature“. The last column holds the information about the sensor used.
- Snowpit overview photos^45^. All photos taken with the Olympus camera (and other digital cameras) are uploaded in jpeg format.
- Snowpit SfM images^46^. The multi-image photogrammetry images are all in jpeg format. The targets can be used to measure the relief of the ice and snow surfaces.
- Snow permittivity^47^. The permittivity, temperature and density measurements are stored in an easy-to-read Excel file.
- Snowpit snow density cutter profiles^48^. Measurements taken with the density cutter are saved in an Excel file. The first three columns give information on the event. The column “Snow weight (cutter)“ contains the weight measured when putting the filled cutter on top of the scale (0 g = Empty cutter on the scale). The snow density is recorded in kg m^−3^. The sensor cutter used (in cm^3^ is specified in the “Sensor cutter” column. The scale used is noted in the column “Sensor scale“. The last column is again for comments.
- Snowpit height measurements^49^. The snow height table contains the device operation ID and total snow height. If total snow height differs locally in one pit, adding several snow heights by repeating the Device ID and putting several snow heights from the same pit under each other is possible. Comments go into the last column.
- Snowpit GPS locations^50^. Waypoints GPX file can be found for most events directly uploaded from the GPS device.
- Snowpit salinity profiles^51^. The salinity was taken alongside the density cutter; therefore, the height of the samples should be comparable. First, the sample was taken and measured for density. The same snow sample was then stored in a plastic container, melted and later analysed for salinity and isotopes. The salinity containers were all labelled. The label goes into the column “Containment“. Salinity is only measured in ppt. Therefore, the red-marked columns were only used for the events labelled PS122/1. Column “Temperature“ contains the temperature by which salinity was measured. After measuring salinity from the A-pits, the melted snow is stored in vials for stable water isotope measurements.
- Snowpit stable water (oxygen and hydrogen) isotope samples^52^. Oxygen *δ*^18^O and Hydrogen *δ*^2^H isotopes taken from the snowpits were analysed at the WSL laboratory in Switzerland.
- Snowpit Micro-Computer Tomograph (Micro-CT) scans^53^. Profiles of density and SSA are published for each sample collected at the snowpit site. The corresponding event ID (or device operation ID) for each sample can be used to construct full profiles of density and SSA for each snowpit site visit.

### Parameter coverage

**Specific surface area:** Micro-CT, SMP, NIR camera.

**Density:** Micro-CT, manual density cutter, SMP.

**Wetness:** denoth probe, dielectric permittivity.

**Snow water equivalent:** ETH SWE tube, density cutter, SMP, Micro-CT.

**Temperature:** Thermometer 1,2,3,4,5.

*δ*^18^**O,** *δ*^2^**H:** Samples collected in the field and analysed at the WSL laboratory at the Swiss Federal Institute for Forest, Snow and Landscape Research.

**Salinity:** Conductivity probe.

**Chemical sampling:** Parameters are not included in this publication.

NOTE: The “Additional metadata” provided in the snowpit metadata publication^38^ can be used to identify which measurement instruments are associated with each snowpit device operation ID.

## Technical Validation

**Fig. 8:**
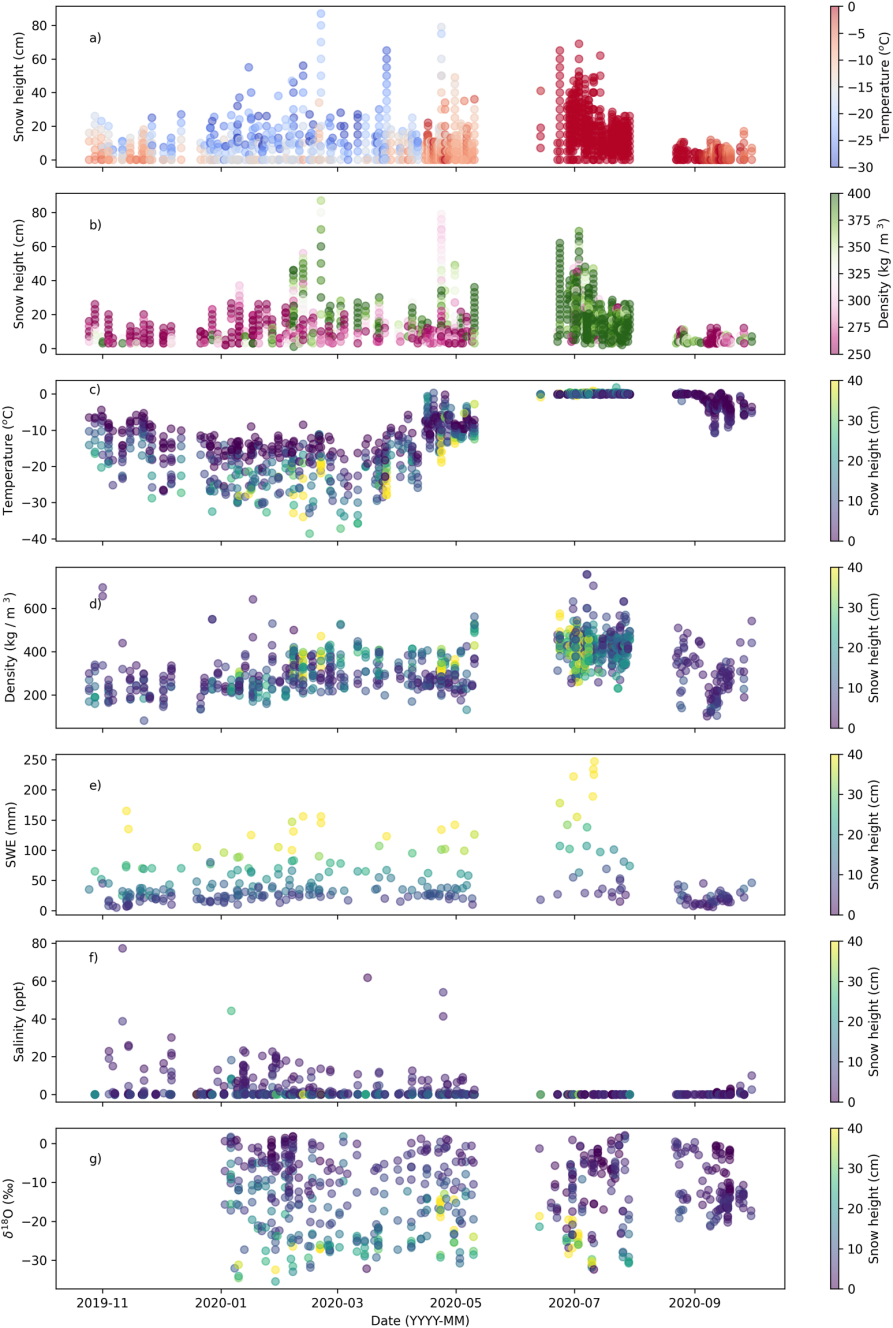
Time series of parameters for the entire season. This figure gives an overview of the published datasets on Pangaea covering the entire season and collected on all three central observatories (CO1, CO2 and CO3). One marker in these graphs indicates one measurement. The marks have transparency, so the darker marks represent multiple measurements at one timestamp. (**a,c**) show a temperature time series taken at different heights in the snowpack. (**b,d**) show measurements of snow density using the density cutter, where one point represents one cutter measurement. (**e**) shows the SWE tube time series, (**f**) shows the salinity time series, and finally, (**g**) shows the stable water isotope *δ*^18^O time series.

Throughout the expedition, instruments were calibrated and the data was quality controlled. Post-processing is not something that is required for many of the variables collected. Figures plotting the temperatures density, SWE and snow height can be used to get a quick overview of the conditions throughout the year. These plots can be seen in Fig. 8, where one point indicates one measurement in the snowpit. These graphs are a combination of multiple snowpit site locations.

**Fig. 9:**
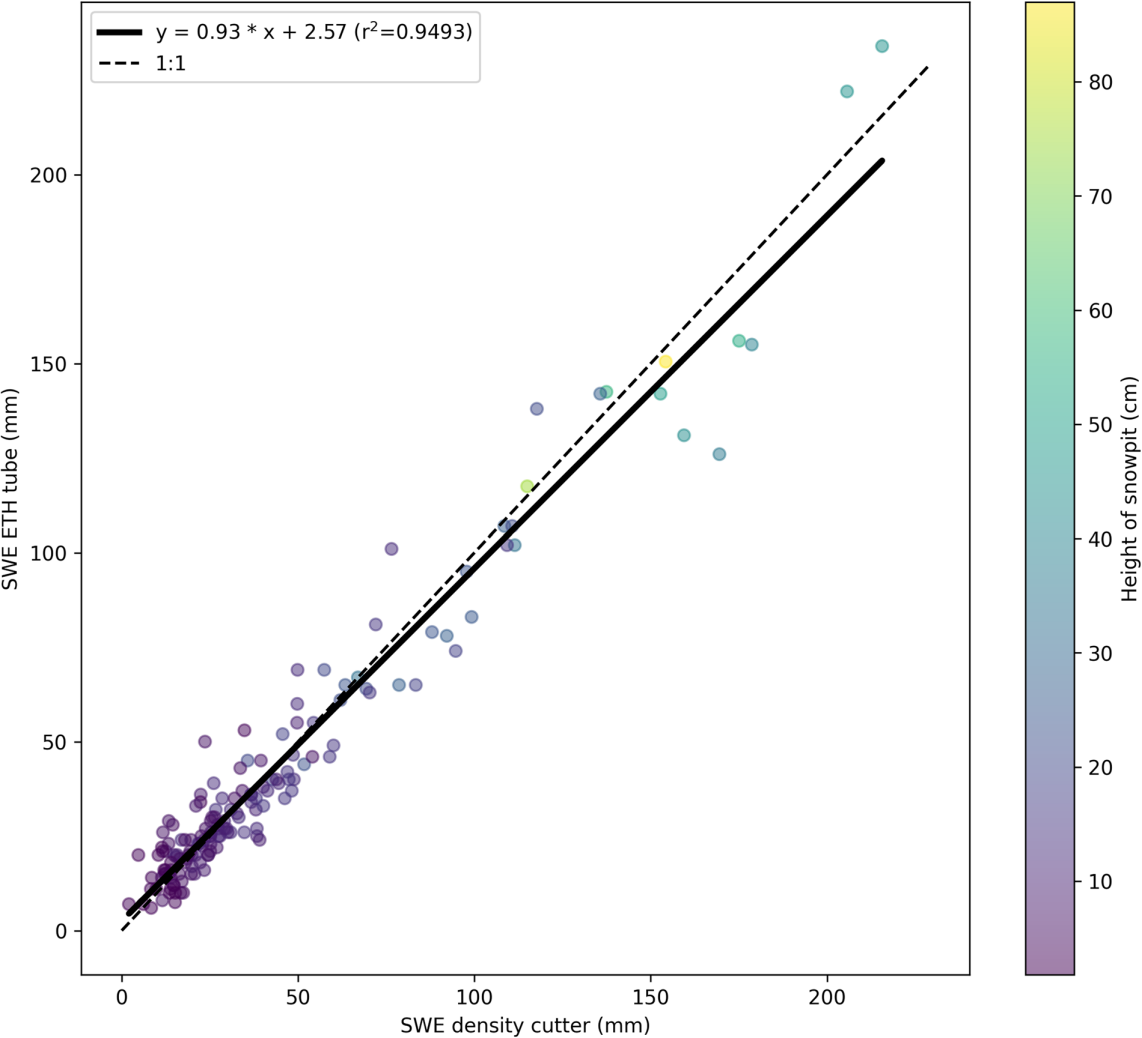
SWE parameter cross-checked against ETH tube measurement and density cutter measurements. At each snowpit, it was common to take measurements of SWE using the aluminium SWE ETH-tube and density using the density cutter. By using the equation linking SWE to the density and volume of snow, we are able to compare the two instruments. This figure presents a SWE comparison of the SWE ETH-tube to the SWE calculated using the density cutter covering measurements of the entire season and collected on all three central observatories (CO1, CO2 and CO3). The average of all density cutter measurements in one profile was multiplied by the corresponding height in the SWE-ETH tube to obtain the SWE for the density cutter. The SWE-ETH tube values were taken directly from the dataset. If there were multiple measurements for one profile, the average SWE was taken.

A SWE comparison of two different methods can be seen in Fig. 9. In this figure, we can see the SWE parameter cross-checked against ETH tube measurement and bulk density cutter measurements. The ETH SWE tube’s values can be seen in the y-axis, and the SWE measured with a density cutter can be seen in the x-axis. The average of all density cutter measurements in one profile was multiplied by the corresponding height in the SWE-ETH tube to obtain the SWE for the density cutter. The SWE-ETH tube values were taken directly from the dataset^43^. The average was taken if there were multiple SWE ETH tube measurements for one profile. The variability in this figure is due to the different volumes being measured. The ETH tube measures the whole snowpack, whereas the density cutter takes the snowpack in 3 cm intervals and therefore has different errors associated with it. Due to the complex nature of the snowpack on sea ice, the layering can be locally highly variable. This may also produce variability in Fig. 9.

**Fig. 10:**
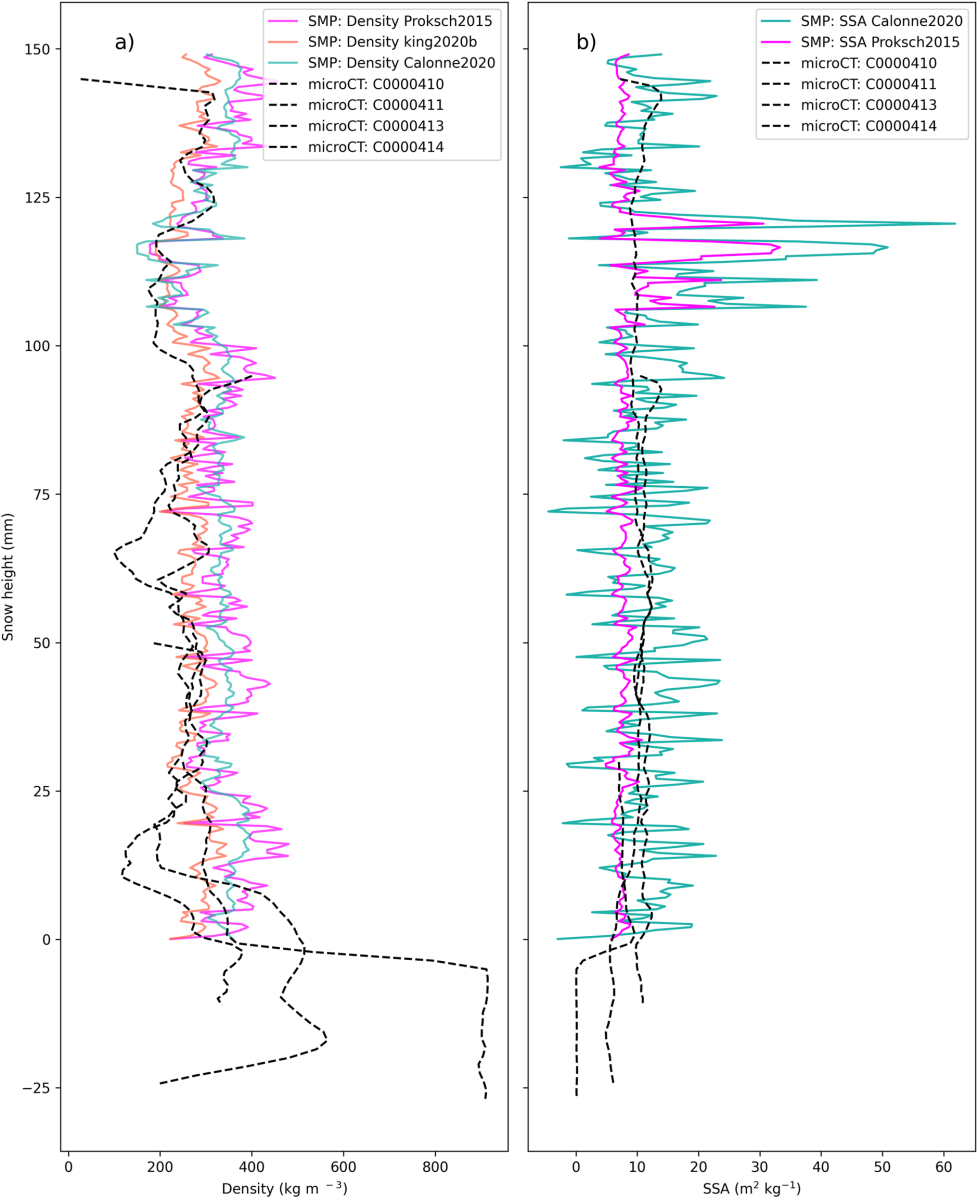
A co-location of SMP and micro-CT measurements for device operation ID PS122/3_38-94. (**a**) Shows density derived from the micro-CT and SMP parameterisations; Proksch2015^23^, King2020b^25^ and Calonne2020^54^. (**b**) shows SSA derived from the micro-CT and SMP parameterisations; Calonne2020^54^ and Proksch2015^23^. This figure highlights the importance of taking care when choosing the density and SSA parameterisations in all future analyses of this dataset.

Figure 10 illustrates how the SMP parameterisations of density and SSA need to be carefully used. Figure 10a shows the full snowpit profiles for density measured using micro-CT samples and SMP density parameterisations^23,25,54^. Figure 10b shows the full snowpit profile for SSA measured by four micro-CT samples taken for device operation ID PS122/3_38–94 and one co-located SMP profile. The SMP penetration resistance profile is used alongside parameterisations^23,54^ to obtain SSA values. The difference between micro-CT and SSA profiles is also influenced by both spatial heterogeneity and the different parameterisations^55^.

## Usage Notes

For specific queries, please direct any questions to;

- David Wagner (2019-10-25 - 2019-12-11, for event IDs starting PS122/1)
- Martin Schneebeli (2019-12-17 - 2020-02-23, for event IDs starting PS122/2)
- Amy Macfarlane (2020-02-25 - 2020-07-30, for event IDs starting PS122/3 and PS122/4)
- Ruzica Dadic (2020-08-21 - 2020-09-30, for event IDs starting PS122/5)
- Aikaterini Tavri (for queries regarding the permittivity dataset)

To download more than one file at a time from Pangaea, please refer to the following link: https://wiki.pangaea.de/wiki/Download_many.

## Data Availability

Due to the format of most of these datasets being Excel files, there is no code published to read these datasets. The SMP dataset^40^ has a custom code to read the pnt files. This can be accessed at https://github.com/slf-dot-ch/snowmicropyn.git. The NIR MAPiR software can be used for post-processing and calibration of NIRbox images. This can be accessed at https://www.mapir.camera/collections/software.

## References

[1] Eicken H, Fischer H, Lemke P (1995). Effects of the snow cover on Antarctic sea ice and potential modulation of its response to climate change. Annals of Glaciology.

[2] Fichefet T, Maqueda M (1999). Modelling the influence of snow accumulation and snow-ice formation on the seasonal cycle of the Antarctic sea-ice cover. Climate Dynamics.

[3] Massom RA (2001). Snow on Antarctic sea ice. Reviews of Geophysics.

[4] Lecomte O, Fichefet T, Flocco D, Schroeder D, Vancoppenolle M (2015). Interactions between wind-blown snow redistribution and melt ponds in a coupled ocean-sea ice model. Ocean Modelling.

[5] Lecomte O (2013). On the formulation of snow thermal conductivity in large-scale sea ice models. Journal of Advances in Modeling Earth Systems.

[6] Sturm, M. & Massom, R. A. Snow in the sea ice system: Friend or foe. *Sea ice* 65–109 (2017).

[7] Granskog MA (2017). Snow contribution to first-year and second-year Arctic sea ice mass balance north of svalbard. Journal of Geophysical Research: Oceans.

[8] Arndt S (2017). Influence of snow depth and surface flooding on light transmission through Antarctic pack ice. Journal of Geophysical Research: Oceans.

[9] Petty AA, Webster M, Boisvert L, Markus T (2018). The NASA eulerian snow on sea ice model (NESOSIM) v1. 0: initial model development and analysis. Geoscientific Model Development.

[10] Webster M (2018). Snow in the changing sea-ice systems. Nature Climate Change.

[11] Sturm M, Holmgren J, Perovich DK (2002). Winter snow cover on the sea ice of the Arctic Ocean at the Surface Heat Budget of the Arctic Ocean (SHEBA): Temporal evolution and spatial variability. Journal of Geophysical Research: Oceans.

[12] Merkouriadi I, Cheng B, Hudson SR, Granskog MA (2020). Effect of frequent winter warming events (storms) and snow on sea-ice growth–a case from the Atlantic sector of the Arctic Ocean during the N-ICE2015 campaign. Annals of Glaciology.

[13] Sankelo P, Haapala J, Heiler I, Rinne E (2010). Melt pond formation and temporal evolution at the drifting station tara during summer 2007. Polar Research.

[14] Radionov, V. F., Bryazgin, N. N. & Alexandrov, E. I. The snow cover of the Arctic basin. Tech. Rep., WASHINGTON UNIV SEATTLE APPLIED PHYSICS LAB (1997).

[15] Nandan V (2017). Effect of snow salinity on cryosat-2 arctic first-year sea ice freeboard measurements. Geophysical Research Letters.

[16] Maksym T (2019). Arctic and Antarctic sea ice change: contrasts, commonalities, and causes. Annual Review of Marine Science.

[17] Meredith, M. *et al*. Polar Regions. Chapter 3, IPCC Special Report on the Ocean and Cryosphere in a Changing Climate. *IPCC, Polar Regions* (2019).

[18] Arrigo, K. R. Sea ice as a habitat for primary producers. *Sea ice* 352–369 (2017).

[19] Light, B., Grenfell, T. C. & Perovich, D. K. Transmission and absorption of solar radiation by Arctic sea ice during the melt season. *Journal of Geophysical Research: Oceans* **113** (2008).

[20] Nicolaus, M. *et al*. Overview of the MOSAiC expedition: Snow and sea ice (2022).

[21] Manninen T (2021). Effect of small-scale snow surface roughness on snow albedo and reflectance. The Cryosphere.

[22] Irvine-Fynn TD, Sanz-Ablanedo E, Rutter N, Smith MW, Chandler JH (2014). Measuring glacier surface roughness using plot-scale, close-range digital photogrammetry. Journal of Glaciology.

[23] Proksch M, Löwe H, Schneebeli M (2015). Density, specific surface area, and correlation length of snow measured by high-resolution penetrometry. Journal of Geophysical Research: Earth Surface.

[24] Schneebeli M, Pielmeier C, Johnson JB (1999). Measuring snow microstructure and hardness using a high resolution penetrometer. Cold Regions Science and Technology.

[25] King J (2020). Local-scale variability of snow density on arctic sea ice. The Cryosphere.

[26] Kaltenborn, J., Clay, V., Macfarlane, A. R. & Schneebeli, M. Machine learning for snow stratigraphy classification. In *NeurIPS 2021 Workshop on Tackling Climate Change with Machine Learning* (2021).

[27] Conger SM, Mcclung DM (2009). Comparison of density cutters for snow profile observations. Journal of Glaciology.

[28] Stevens. Stevens ® Water Monitoring System, Inc. The Hydra Probe ® Soil Sensor Comprehensive Stevens Hydra Probe Users Manual (2015).

[29] Geldsetzer T, Langlois A, Yackel J (2009). Dielectric properties of brine-wetted snow on first-year sea ice. Cold Regions Science and Technology.

[30] Backstrom LG, Eicken H (2006). Capacitance probe measurements of brine volume and bulk salinity in first-year sea ice. Cold regions science and technology.

[31] Scharien, R. K., Geldsetzer, T., Barber, D. G., Yackel, J. J. & Langlois, A. Physical, dielectric, and C band microwave scattering properties of first-year sea ice during advanced melt. *Journal of Geophysical Research: Oceans* **115** (2010).

[32] Survey3N Camera - Near Infrared (NIR) - MAPIR CAMERA. https://www.mapir.camera/products/survey3n-camera-near-infrared-nir.

[33] Matzl M, Schneebeli M (2006). Measuring specific surface area of snow by near-infrared photography. Journal of Glaciology.

[34] Scanco Medical AG. µCT 90 desktop microCT scanner. https://www.scanco.ch/microct90.html (2022).

[35] YSI incorporated. Ysi model 30 ysi model 30m handheld salinity, conductivity and temperature system operations manual. https://www.ysi.com/File%20Library/Documents/Manuals%20for%20Discontinued%20Products/030136-YSI-Model-30-Operations-Manual-RevE.pdf (2007).

[36] Itkin P (2021). PANGAEA.

[37] Nicolaus M (2021). PANGAEA.

[38] Macfarlane AR (2021). PANGAEA.

[39] Macfarlane AR (2022). PANGAEA.

[40] Macfarlane AR (2021). PANGAEA.

[41] Macfarlane AR (2022). PANGAEA.

[42] Macfarlane AR (2022). PANGAEA.

[43] Macfarlane AR (2022). PANGAEA.

[44] Macfarlane AR (2022). PANGAEA.

[45] Macfarlane AR (2022). PANGAEA.

[46] Macfarlane AR (2021). PANGAEA.

[47] Macfarlane AR (2022). PANGAEA.

[48] Macfarlane AR (2022). PANGAEA.

[49] Macfarlane AR (2022). PANGAEA.

[50] Macfarlane AR (2021). PANGAEA.

[51] Macfarlane AR (2022). PANGAEA.

[52] Macfarlane AR (2022). PANGAEA.

[53] Macfarlane AR (2022). PANGAEA.

[54] Calonne N (2020). The RHOSSA campaign: multi-resolution monitoring of the seasonal evolution of the structure and mechanical stability of an alpine snowpack. The Cryosphere.

[55] Löwe H, Riche F, Schneebeli M (2013). A general treatment of snow microstructure exemplified by an improved relation for thermal conductivity. The Cryosphere.

[56] Knust R (2017). Polar research and supply vessel POLARSTERN operated by the Alfred-Wegener-Institute. Journal of large-scale research facilities JLSRF.

[57] Nixdorf, U. *et al*. MOSAiC extended acknowledgement. *Zenodo* (2021).

