# Author Correction: A Database of Snow on Sea Ice in the Central Arctic Collected during the MOSAiC expedition

**DOI:** 10.1038/s41597-023-02413-7

**Published:** 2023-07-28

**Authors:** Amy R. Macfarlane, Martin Schneebeli, Ruzica Dadic, Aikaterini Tavri, Antonia Immerz, Chris Polashenski, Daniela Krampe, David Clemens-Sewall, David N. Wagner, Donald K. Perovich, Hannula Henna-Reetta, Ian Raphael, Ilkka Matero, Julia Regnery, Madison M. Smith, Marcel Nicolaus, Matthias Jaggi, Marc Oggier, Melinda A. Webster, Michael Lehning, Nikolai Kolabutin, Polona Itkin, Reza Naderpour, Roberta Pirazzini, Stefan Hämmerle, Stefanie Arndt, Steven Fons

**Affiliations:** 1 WSL Institute for Snow and Avalanche Research SLF, Davos Dorf, Switzerland; 2 Victoria University of Wellington, Wellington, New Zealand; 3 Department of Geography, University of Victoria, Victoria, BC Canada; 4 Alfred-Wegener-Institut Helmholtz-Zenütrum fr Polar und Meeresforschung, Bremerhaven, Germany; 5 Thayer School of Engineering at Dartmouth College, Hanover, New Hampshire USA; 6 CRYOS, School of Architecture, Civil and Environmental Engineering, EPFL, Lausanne, Switzerland; 7 Finnish Meteorological Institute, Helsinki, Finland; 8 Svalbard Integrated Arctic Earth Observing System, P.O. Box 156, 9171 Longyearbyen, Norway; 9 Woods Hole Oceanographic Institution, Woods Hole, Massachusetts USA; 10 International Arctic Research Center, University of Alaska Fairbanks, Fairbanks, USA; 11 Arctic and Antarctic Research Institute, AARI, Saint-Petersburg, Russia; 12 UiT The Arctic University of Norway, Tromsø, Norway; 13 SCANCO medical AG, Wangen-Brüttisellen, Switzerland; 14 Cryospheric Sciences Lab, NASA Goddard Space Flight Center, Greenbelt, Maryland USA; 15 Earth System Science Interdisciplinary Center, University of Maryland, College Park, Maryland USA

**Keywords:** Cryospheric science, Techniques and instrumentation, Cryospheric science, Techniques and instrumentation

Correction to: *Scientific Data* 10.1038/s41597-023-02273-1, published online 22 June 2023

The original version showed the wrong image for Figure 3, with the image for Figure 4 used for both. This has been corrected in the pdf and HTML versions of the article, with the correct version of Figure 3 replacing the duplicated figure. The dates in the figure captions were also incorrect and have been amended as follows:

Figure 3 caption: “from 2019-10-25 - 2020-07-30” modified to “from 2019-10-25 - 2020-05-15”

Figure 4 caption: “from 2020-02-25 - 2020-07-30” modified to “from 2020-06-13 - 2020-07-30”

